# The Efficacy of Endoscopic Papillary Balloon Dilation for Patients with Acute Biliary Pancreatitis

**DOI:** 10.1155/2015/575898

**Published:** 2015-04-09

**Authors:** Wei-Chih Sun, Hoi-Hung Chan, Kwok-Hung Lai, Tzung-Jiun Tsai, Huey-Shyan Lin, Kung-Hung Lin, Kai-Ming Wang, Sung-Shuo Kao, Po-Hung Chiang, Jin-Shiung Cheng, Ping-I Hsu, Wei-Lun Tsai, Wen-Chi Chen, Yun-Da Li, E-Ming Wang

**Affiliations:** ^1^Division of Gastroenterology, Department of Internal Medicine, Kaohsiung Veterans General Hospital, Kaohsiung 813, Taiwan; ^2^School of Medicine, National Yang-Ming University, Taipei 112, Taiwan; ^3^Department of Biological Sciences, National Sun Yat-sen University, Kaohsiung 804, Taiwan; ^4^College of Pharmacy and Health Care, Tajen University, Pingtung 907, Taiwan; ^5^Department of Health-Business Administration, Fooyin University, Kaohsiung 831, Taiwan

## Abstract

*Background*. No study investigated the efficacy and safety of endoscopic papillary balloon dilation (EPBD) for the treatment of acute biliary pancreatitis (ABP).* Method*. We retrospectively reviewed the effects of EPBD on patients with ABP from February 2003 to December 2012. The general data, findings of image studies, details of the procedure, and outcomes after EPBD were analyzed.* Result*. Total 183 patients (male/female: 110/73) were enrolled. The mean age was 65.9 years. Among them, 155 patients had mild pancreatitis. The meantime from admission to EPBD was 3.3 days. Cholangiogram revealed filling defects inside the common bile duct (CBD) in 149 patients. The mean dilating balloon size was 10.5 mm and mean duration of the dilating procedure was 4.3 minutes. Overall, 124 patients had gross stones retrieved from CBD. Four (2.2%) adverse events and 2 (1.1%) intraprocedure bleeding incidents but no procedure-related mortality were noted. Bilirubin and amylase levels significantly decreased after EPBD. On average, patients resumed oral intake within 1.4 days. The clinical parameters and outcomes were similar in patients with different severity of pancreatitis.* Conclusion*. EPBD can be effective and safe for the treatment of ABP, even in patients presenting with severe disease.

## 1. Introduction

Acute biliary pancreatitis (ABP) is a common disease with an annual incidence of 4.9–80.0/100,000 [1]. In the United States and Western Europe, 35–60% of the patients with acute pancreatitis resulted from biliary stone [2–4]. Although most of the cases with ABP are mild and self-limited, there are 20% of patients develop severe complications such as necrotizing pancreatitis and/or multiple organ failure with a mortality rate of 13–50% [2–5]. The pathogenesis of ABP is complex, and the main factor is transient or persistent ampullary obstruction by gallstones [6–9]. Prolonged obstruction will aggravate pancreatic inflammation and contribute to severe pancreatitis [8]. Previous animal and human studies suggested that duration of obstruction over 48 hours may probably result in pancreatic necrosis [10–13]. With regard to this point of view, any measure that can relieve ampullary obstruction as soon as possible is a crucial part in minimizing the subsequent local or systemic complications [14].

Surgical removal of bile duct stones has been first advocated in 1978; however, the associated mortality was unacceptably high and up to 67% in a randomized trial [15, 16]. Sphincterotomy (EST) for the treatment of ABP was reported by Safrany et al. in 1980, with regard to its nature of less invasiveness than that of surgery [17]. Current guidelines recommend emergency EST in the treatment of patients with ABP with concomitant cholangitis and/or persistent biliary obstruction regardless of the predicted severity of pancreatitis [18–21]. On the other hand, endoscopic papillary balloon dilation (EPBD), which is easy to perform using the wire-guided method, has become an alternative to EST for the treatment of CBDS since its first introduction by Staritz et al. in 1982 [22]. Although EPBD is not commonly used in western countries due to the consideration of post-ERCP pancreatitis, it is widely used as EST in Asian countries with good efficacy [23]. Nevertheless, no study assessed the effects of EPBD in patients with ABP. In order to investigate the efficacy and safety of EPBD in the treatment of ABP, we conducted a retrospective study to share the experience in our hospital.

## 2. Materials and Methods

### 2.1. Patients

Between February 2003 and December 2012, consecutive patients with ABP successfully managed by ERCP/EPBD in Kaohsiung Veterans General Hospital were reviewed. The successful EPBD was defined as a complete performance of balloon dilation and the subsequent stone extraction procedures with clearance of bile duct (no gross stone was found in cholangiography) within two endoscopic sessions. Total 500 patients were diagnosed of acute biliary pancreatitis. Among them, three hundred and seventeen patients who did not receive EPBD were excluded from the study, which include 25 contraindications with/without emergency percutaneous drainage, 30 refusals of ERCP, 96 cases of spontaneous stone pass-out before ERCP, 24 failures of ERCP, 93 cases of diagnostic ERCP only, 48 cases of EST, and one failure of EPBD.

Diagnosis of acute pancreatitis was established when fulfilling any two of the following three criteria: (1) typical abdominal pain (acute onset of a persistent, severe, epigastric pain often radiating to back); (2) serum amylase or lipase levels higher than three times the upper limit of normal; (3) characteristic findings of acute pancreatitis in abdominal ultrasound or contrast-enhanced computed tomography (CECT) [18, 19, 24, 25]. A biliary etiology was based on the exclusion of alcoholic or metabolic causes of pancreatitis and the presence of at least one of the following criteria: (1) gallbladder stone or sludge found by ultrasound or CECT; (2) dilated common bile duct (CBD) found by ultrasound or CECT (diameter > 7 mm if gallbladder is intact and diameter > 11 mm if gallbladder has been removed); (3) abnormal liver biochemistries including alanine aminotransferase (ALT) > 40 U/L, alkaline phosphatase (Alk-P) > 128 U/L, *γ*-glutamyltransferase (*γ*-GT) > 60 U/L, or an elevated serum bilirubin level >1.6 mg/dL. Severe pancreatitis was defined as Ranson's score ≧ 3 and/or CT severity index > 3 [26, 27]. In addition, acute cholangitis was defined by Charcot's triad (fever > 38.5°C, epigastric pain, and jaundice) or signs of sepsis [14].

Some meta-analyses did not suggest emergency ERCP for mild pancreatitis [28–30], so patients were stratified into group A (mild pancreatitis) and group B (severe pancreatitis) for further comparison of clinical outcomes and to determine which one was beneficial to the endoscopic procedure.

### 2.2. Timing of Endoscopic Treatment

Currently, all guidelines recommended an emergency ERCP in patients with ABP with coexisting cholangitis and/or persistent biliary obstruction, but the optimal timing for ERCP differed among the guidelines: within 72 hours after onset of symptoms (World Congress of Gastroenterology, American Thoracic Society, British Society of Gastroenterology, Dutch Society of Internal Medicine), within 24 hours after hospital admission (German and American College of Gastroenterology), or controversial (International Association of Pancreatology, American Gastroenterological Association, Japanese Guidelines) [31]. In our study, when patients had either concomitant cholangitis or persistent biliary obstruction with the presentation of Charcot's triad, bacteremia, progressive abdominal pain, or deterioration of liver biochemistries, we tended to perform ERCP as early (within 72 hours after hospital admission) as possible.

### 2.3. Endoscopic Procedures

Patients were conscious for this procedure and received 10% xylocaine spray for local anesthesia of the pharynx, intramuscular injection with 40 mg hyoscine-*N*-butylbromide, and intramuscular injection with 25–50 mg meperidine. ERCP was performed in the standard manner using a side-view endoscope (JF-240; Olympus Optical Corporation, Tokyo, Japan). After selective cannulation of the common bile duct by the catheter, cholangiography was performed to evaluate the size of CBD, presence of filling defects inside CBD, and size of CBD filling defects. A 0.035-inch guide wire (Boston Scientific, Corp, MA, USA) was then inserted into the bile duct through the catheter. A dilating balloon (CRE balloon 5.5 cm in length, 6–8 mm/8–10 mm/10–12 mm/12–15 mm in diameter; Boston Scientific, Corp, Ireland) was passed via the prepositioned 0.035-inch guide wire into the bile duct. Using fluoroscopic and endoscopic guidance, the balloon was inflated with sterile saline solution up to the optimal size (at least > six mm in diameter) and duration (from 1.5 to 5 minutes) according to the patients' condition and tolerance. In order to minimize the risk of perforation, the size of the balloon should be not exceed the size of the CBD. After the balloon and guide wire were removed, the CBDS was retrieved out using a Dormia basket or balloon-tipped catheter with or without the aid of mechanical lithotripsy (BML-4Q; Olympus Optical, Tokyo, Japan). Unnecessary cannulation or contrast injection of pancreatic duct was avoided. A second attempt of stone extraction was performed within three days if there was incomplete removal of stones in the first treatment session. All the patients were observed in the hospital for at least 24 hours after endoscopic treatment.

### 2.4. Assessments and Outcomes

Demographic data of patients and hospital course was collected from clinical records, including presence of juxtapapillary diverticula (JPD), pancreatic duct enhancement, CBD diameter, number and size of stones, size of dilating balloon, and dilating duration, and presence of extracted stone was recorded. Successful bile duct clearance was defined as complete if the final cholangiogram revealed no more filling defects. The day after the endoscopic procedure, a blood sample for measurement of serum amylase and total bilirubin was obtained. ERCP/EPBD related adverse events were recorded according to the definitions and grading systems from the consensus of an American Society of Gastrointestinal Endoscopy Workshop [32]. The definition for exacerbation of pancreatitis after ERCP is as follows: (1) new or worsened abdominal pain, (2) rise of serum amylase at least three times above the upper limit of normal at 24 hours after ERCP, (3) requiring at least 2 days of hospitalization (2-3 days: mild degree; 4–10 days: moderate degree; more than 10 days: severe degree). The definition for post-ERCP cholangitis is (1) fever > 38.5°C and (2) persistent cholestasis more than 24 hours. The definition for post-ERCP cholecystitis is (1) newly developed pain and tenderness in RUQ and (2) image of gallbladder wall thickening and pericholecystic fluid. The definition for post-ERCP bleeding is hematemesis and/or melena with a hemoglobin decrease of at least 2 g/dL or the need for blood transfusion. Time from admission to ERCP/EPBD, time to resume oral intake after ERCP/EPBD, total hospital days, incidence of ERCP/EPBD related adverse events, and evolutions of laboratory data were assessed to measure the clinical effect and safety of ERCP/EPBD on patients with ABP.

### 2.5. Ethics Statement

This study was approved by the Ethics Committee and the Institutional Review Board of the Kaohsiung Veterans General Hospital (VGHKS13-CT6-12). This is a retrospective study that did not involve patient intervention or the need for obtaining clinical specimens, and all the data were analyzed anonymously. Therefore, informed consent was waived. The waiving of informed consent was approved by the Kaohsiung Veterans General Hospital Institutional Review Board.

### 2.6. Statistical Analysis

All statistical analyses were performed using the PASW statistics (IBM, New York, NY, USA). The continuous valuables are expressed as mean ± SD. Pearson Chi-square analysis or Fisher's exact test was used for the comparison of categorical variables, while continuous variables were compared using the paired-sample and independent-sample *t*-tests. Multivariable logistic regression was used to find out the possible predictors for CBD stones. A two-tailed *P* value of <0.05 was considered significant in all tests.

## 3. Results

Within the study period, a total of 183 patients were enrolled in this retrospective study. Characteristics of overall patients were shown in Table 1. There were 110 (60.7%) males and 73 (39.3%) females. The mean age of the patients was 65.9 years. 155 (84.7%) patients were diagnosed as mild pancreatitis and the rest of 28 (15.3%) were severe in degree. Radiological examinations revealed the gallbladder in situ in 165 (90.2%) patients (147 had gallbladder stones) and presence of CBD stones in 91 (49.7%) patients. The results of EPBD and clinical outcomes were shown in Table 2. The meantime from admission to receiving EPBD was 3.3 days. Cholangiogram revealed dilated CBD in 159 (86.9%) patients with mean CBD size of 11.8 ± 4.2 mm. There was positive filling defects inside CBD in 149 (81.4%) patients, and the mean filling defect size was 6.8 ± 4.6 mm. The mean size of dilating balloon was 10.5 ± 1.8 mm and mean duration of the dilating procedure was 4.3 ± 1.1 minutes. There were gross stones retrieved from CBD in 124 (67.8%) patients. The endoscopic treatment was successful in the first session of 179 (97.8%) patients. Four (2.2%) patients had large CBD stones (>1.5 cm) (2 of them received mechanical lithotripsy in first session), whom required second endoscopic treatment to remove residual CBD stones within three days. The rate of positive pancreatic duct injection was 50.8% of patients, and it was significantly higher in the patients with severe pancreatitis than in those with mild pancreatitis (67.9% versus 47.7%, *P* = 0.05). There were 5 (2.7%) procedure-related adverse events, including 3 mild pancreatitis, 1 cholangitis, and 1 cholecystitis. All the complications could be controlled by conservative treatment and no procedural mortality was noted. EPBD caused the reduction of serum amylase in 90.7% of patients and reduction of serum total bilirubin in 83.1% of patients. On average, time to resume oral intake after ERCP was 1.4 days. The average hospital day was 9.1 and 9.6 for patients with mild and severe pancreatitis, respectively.

Patients were divided into two groups: (A) 155 patients with mild pancreatitis and (B) 28 patients with severe pancreatitis. Baseline data of the two groups of patients were shown in Table 1. Significant differences in serum level of WBC, blood sugar, amylase, and borderline significant differences in age and serum total bilirubin level were found between the two groups. There were significant difference in size of dilating balloon and borderline difference in pancreatic duct enhancement (Table 2). Other clinical outcomes were similar between the two groups. The evolution of serum total bilirubin and serum amylase level by EPBD in overall patients and between patients with the mild and severe degree of acute biliary pancreatitis was shown in Table 3. Between the two groups, the levels of serum total bilirubin were significantly different before EPBD but there was no significant difference after EPBD. The levels of serum total bilirubin were significantly decreased in overall patients, regardless of the severity of pancreatitis. Although the levels of serum amylase were significantly decreased after EPBD in overall patients, there were no such similar differences in the reduction of serum amylase between the two groups. Moreover, the levels of serum amylase before and after EPBD were not significantly different between the two groups. Multivariable logistic regression analysis revealed that old age and high serum bilirubin level before ERCP are significant predictors for gross stone retrieved during the ERCP/EPBD procedures (Table 3).

## 4. Discussion

Since the introduction of endoscopic treatment of ABP, it has been proved to be beneficial to the outcome of the disease [33]. Although EPBD has been shown to be an alternative to EST to remove the common bile duct stones with a similar success rate and lower risk of immediate complications such as bleeding or perforation [32–38], the clinical effect of EPBD for the treatment of ABP is rarely reported due to the fact that the side effect of pancreatitis was emphasized before. In fact, in this study, the incidence of overall adverse events was only 2.2% and all patients recovered after conservative treatment. Regarding the literature, although there is no difference in overall complication rates between EST and EPBD, a higher risk of post-ERCP pancreatitis was reported in some studies [39, 40]. Certain predictive factors were identified for the development of post-ERCP pancreatitis such as female gender, difficult cannulation, pancreatic duct injection, and normal serum total bilirubin level [41–43]. In this study, most patients (60.7%) were males and 81.9% had an elevated serum total bilirubin level. Superfluous injection of contrast medium into the pancreatic duct is certainly considered to lead to increasing the risk of pancreatitis. Although the considerable amount of our patients had encountered pancreatic duct injection, the volume of contrast medium injection was quietly minimized. Once the head portion of pancreatic duct filled with contrast, we would stop injection immediately and withdraw the catheter in order to minimize the parenchymal injury. Moreover, from the latest reports in recent 5 years [39, 44–50], pancreatitis more frequently developed in the patients using the small balloon (8 mm) and short duration (<3 min) than the patients using the large balloon and long duration. In the report of randomized trial from Liao et al. [51], compared with conventional 1-minute EPBD, 5-minute EPBD improves efficacy of stone extraction and reduces the risk of pancreatitis. A meta-analysis also demonstrated the duration of EPBD is inversely associated with pancreatitis risk [52]. Besides, long EPBD can adequately loosen the intact sphincter; the widely opened papillary orifice may facilitate the insertion of accessory instruments into the bile duct and decrease the injury of the pancreas [36, 53]. In this study, the mean dilating procedure duration was 4.3 minutes and the mean balloon size was 10.5 mm, and the aforementioned reasons might explain why the incidence of pancreatitis after EPBD was low and only 9.9% patients had hyperamylasemia.

Biliary decompression theoretically ceases progression of biliary pancreatitis and reduces further complications. However, there is still lack of consensus on the role of endoscopic treatment for ABP with regard to the predicted severity of pancreatitis [31]. In some meta-analysis and systemic review studies, early (≦72 hours after admission) routine treatment in mild pancreatitis without concomitant cholangitis or biliary obstruction did not affect the disease course and even causes more mortality [28, 54]. In our study, although most of our patients had mild degree pancreatitis (84.7%), the clinical outcomes (improvement of pancreatitis and the occurrence of complications) of treatment by EPBD were not significantly different between the two groups. There was a trend that we tended to arrange endoscopic treatment sooner for group B (meantime from admission to ERCP: 2.8 days) than group A (meantime from admission to ERCP: 3.4 days) (*P* = 0.08). There was no significant difference in the rates of procedure-related adverse events (A: 1.9% versus B: 3.6%, *P* = 0.49) and intraprocedure bleeding (A: 1.3% versus B: 0.0%, *P* = 0.72). In fact, evolutions of laboratory data, including serum amylase level, serum bilirubin level, time from EPBD to resume oral intake (subside of abdominal pain), and total hospital day were similar regardless of severity of pancreatitis.

A small gallstone impacted in the common bile duct was recorded in 26% to 72% of ABP patients when receiving operation in the early phase, but less than 10% of patients received operation [15, 55]. Spontaneous pass-out of the bile duct stones was reported in 71% to 88% of cases within 48 hours after the onset of ABP [8, 10]. Our results showed that overall 68.4% patients had gross stone retrieved from CBD when EPBD was performed with an average of 3.3 days since admission. It is true that MRCP (or even endoscopic ultrasonography and intraductal ultrasonography) can improve the diagnostic accuracy of small bile duct stones and avoid unnecessary ERCP in patients with ABP [56–61]. However, these diagnostic tools are time-consuming and not always available in our institution. In this study, all patients were clinically prone to require the ERCP intervention as soon as possible; so, MRCP was not routinely arranged in this situation. Indeed, we might perform unnecessary therapeutic ERCP in some patients whose stone had already passed out spontaneously, but this procedure was believed to be useful in preventing recurrent pancreatitis. Endoscopic sphincteroplasty may reduce a 29% to 67% risk of recurrent biliary events even in the patients without gross stone during ERCP in some reports, but it is still controversial whether this invasive procedure should be a routine procedure in those patients or not [62, 63]. Although high predictive values of biochemical markers for CBD stones such as serum total bilirubin (especially greater than 4 mg/dL) and rising liver biochemistries in patients with ABP have been reported by several studies [64–66], multivariate analysis in our study revealed that old age and high serum bilirubin level at the day before EPBD can be helpful in predicting gross stones retrieved from CBD.

There are some limitations of this study associated with retrospective research which include unequal numbers between patients with mild and severe pancreatitis and lack of well-defined optimal timing for EPBD after admission. Further randomized prospective studies may be needed to support the true efficacy of EPBD and figure out who is the best candidate and when is the best timing to receive EPBD for treatment of acute biliary pancreatitis.

## 5. Conclusion

Endoscopic papillary balloon dilation is effective and safe for the treatment of acute biliary pancreatitis, even in the patients presenting with severe disease.

## Figures and Tables

**Table 1:** Characteristics of overall patients and comparisons between patients with mild (A) and severe (B) degree of acute biliary pancreatitis.

Characteristics	Overall (*n* = 183)	Group A (*n* = 155)	Group B (*n* = 28)	*P* value
Gender (male/female)	110/73	93/62	17/11	0.94
Age (mean ± SD, years)	65.9 ± 17.5	66.9 ± 17.3	60.3 ± 17.6	0.06
Body mass index	24.7 ± 3.6	24.7 ± 3.5	24.9 ± 3.9	0.71
Symptom				
Fever	44	34	10	0.09
Nausea and vomiting	111	94	17	0.99
Abdominal pain	103	85	18	0.35
Jaundice	145	119	26	0.12
Laboratory data				
WBC (×10^3^ cu mm^−1^)	11.7 ± 4.4	11.3 ± 4.4	13.6 ± 3.9	0.01
Platelet (×10^3^ cu mm^−1^)	196 ± 66	192 ± 67	217 ± 58	0.09
INR	1.04 ± 0.13	1.06 ± 0.14	1.04 ± 0.11	0.37
AST/ALT (IU/L)	264 ± 443/275 ± 295	262 ± 477/261 ± 304	273 ± 170/351 ± 229	0.84/0.14
Alk-P/*γ*-GT (IU/L)	200 ± 142/479 ± 418	200 ± 145/459 ± 398	200 ± 126/595 ± 519	0.99/0.25
Total bilirubin (mg/dL)	3.7 ± 2.7	3.8 ± 2.8	3.2 ± 1.5	0.09
LDH (IU/L)	311 ± 177	303 ± 179	349 ± 164	0.23
Blood sugar (mg/dL)	159 ± 67	154 ± 62	190 ± 84	0.04
Amylase (IU/L)	1351 ± 1309	1176 ± 1188	2302 ± 1539	<0.01
Lipase (IU/L)	12310 ± 13871	11419 ± 13535	16798 ± 14918	0.09
Radiological finding				
Gallbladder in situ	165	140 (90.3%)	25 (89.3%)	0.54
Gallbladder stone	147	126	21	
Dilated CBD	121	104 (67.1%)	17 (60.7%)	0.51
CBD stone	91	77 (49.7%)	14 (50.0%)	0.98

SD, standard deviation; WBC, white blood cell; INR, international normalized ratio; AST, aspartate transaminase; ALT, alanine transaminase; Alk-P, alkaline phosphatase; *γ*-GT, *γ*-glutamyltransferase; LDH, lactic dehydrogenase; CBD, common bile duct.

**Table 2:** Results of EPBD and clinical parameters in overall patients and comparisons between mild (A) and severe (B) degree of acute biliary pancreatitis.

Results of EPBD	Overall (*n* = 183)	Group A (*n* = 155)	Group B (*n* = 28)	*P* value
Time from admission to initial ERCP (days)	3.3 ± 2.4	3.4 ± 2.5	2.8 ± 1.5	0.08
Juxtapapillary diverticulum	55 (30.1%)	49 (31.6%)	6 (21.4%)	0.28
Dilated CBD	159 (86.9%)	136 (87.7%)	23 (82.1%)	0.42
Mean size of CBD (mm)	11.8 ± 4.2	11.9 ± 4.0	11.5 ± 5.0	0.75
Positive filling defects within CBD	149 (81.4%)	130 (83.9%)	19 (67.9%)	0.23
Mean size of CBD filling defects (mm)	6.8 ± 4.6	6.9 ± 4.7	6.0 ± 3.3	0.14
Dilating balloon size (mm)	10.5 ± 1.8	10.6 ± 1.9	9.9 ± 1.3	0.04
Dilating procedure duration (min)	4.3 ± 1.1	4.3 ± 1.1	4.3 ± 1.0	0.95
Gross stone retrieved from CBD	124 (67.8%)	108 (69.7%)	16 (57.1%)	0.19
Pancreatic duct injection	93 (50.8%)	74 (47.7%)	19 (67.9%)	0.05
Number of mechanical lithotripsies	2 (1.1%)	2 (1.3%)	0 (0.0%)	0.72
Treatment success				
First session	179 (97.8%)	151 (97.4%)	28 (100%)	0.51
Second session	4	4	0	

Procedure-related adverse events	4 (2.2%)	3 (1.9%)	1 (3.6%)	0.49
Exacerbation of pancreatitis	2	1	1	
Cholangitis	1	1	0	
Cholecystitis	1	1	0	
Intraprocedure bleeding	2 (1.1%)	2 (1.3%)	0 (0.0%)	0.72
Evolution of laboratory data after EPBD				
Amylase: increase/decrease	17 (9%)/166 (91%)	15 (10%)/140 (90%)	2 (7%)/26 (93%)	0.67
Total bilirubin: increase/decrease	31 (17%)/152 (83%)	28 (18%)/127 (82%)	3 (11%)/25 (89%)	0.34
Time to resume oral intake after EPBD (days)	1.4 ± 0.9	1.4 ± 0.9	1.5 ± 0.9	0.87
Total hospital day (days)	9.2 ± 4.5	9.1 ± 4.5	9.6 ± 4.3	0.63

EPBD, endoscopic papillary balloon dilation; ERCP, endoscopic retrograde cholangiopancreatography; CBD, common bile duct.

**Table 3:** Predictive factors of gross stone retrieved from common bile duct.

Predicted factor	Univariate	*P* value	Multivariate	*P* value
	HR (95% CI)		HR (95% CI)	
Age (years)	1.019 (1.001–1.037)	0.038	1.024 (1.00–1.048)	0.049^*^
Sex: male	1.758 (0.937–3.297)	0.079	1.676 (0.777–3.614)	0.188
Body mass index	1.048 (0.951–1.156)	0.343	1.047 (0.943–1.164)	0.389
Severity of pancreatitis	0.580 (0.255–1.322)	0.195	0.910 (0.330–2.510)	0.855
CBD stone in CT or ultrasound	1.034 (0.556–1.923)	0.915	0.900 (0.420–1.929)	0.787
CBD filling defects in cholangiogram	1.638 (0.762–3.522)	0.207	1.748 (0.678–4.510)	0.248
T.bil before ERCP	1.248 (1.062–1.467)	0.007	1.311 (1.039–1.655)	0.023^*^
Amylase before ERCP	1.000 (1.000–1.000)	0.490	1.000 (0.999–1.000)	0.253
Concomitant cholangitis	1.306 (0.615–2.772)	0.488	1.565 (0.574–4.267)	0.382

HR, hazard ratio; CBD, common bile duct; CT, computed tomography; T.bil, total bilirubin; ERCP, endoscopic retrograde cholangiopancreatography.

^*^ *P* value < 0.05.
